# Significant CircRNAs in liver cancer stem cell exosomes: mediator of malignant propagation in liver cancer?

**DOI:** 10.1186/s12943-023-01891-y

**Published:** 2023-12-05

**Authors:** Tao Han, Lujun Chen, Kerui Li, Qilin Hu, Yue Zhang, Xuan You, Lei Han, Tingsong Chen, Kai Li

**Affiliations:** 1 Department of Medical Oncology, the First Hospital of China Medical University, 155 Nanjing North Street, Heping District, Shenyang, Liaoning Province 110001 China; 2 Postgraduate College, China Medical University, 77 Puhe Road, North New District, Shenyang, Liaoning Province 110122 China; 3 Department of Medical Oncology, General Hospital of Northern Theater Command, 83 Wenhua Road, Shenhe District, Shenyang, Liaoning Province 110017 China; 4 Department of Oncology, Seventh People’s Hospital of Shanghai University of Traditional Chinese Medicine, No. 358 Datong Road, Pudong New Area, Shanghai, 200137 China; 5 Department of Hepatobiliary Surgery, General Hospital of Northern Theater Command, 83 Wenhua Road, Shenhe District, Shenyang, Liaoning Province 110017 China; 6 Department of Gastrointestinal Oncology Surgery, the First Hospital of China Medical University, 155 Nanjing North Street, Heping District, Shenyang, Liaoning Province 110001 China

**Keywords:** Liver cancer stem cells, Exosomes, circRNA, CD133, Bioinformatics analysis

## Abstract

Hepatocellular carcinoma (HCC), one of the most prevalent forms of cancer worldwide, presents a significant global healthcare challenge. Cancer stem cells (CSCs), which can influence neighboring non-CSCs, are believed to play a crucial role in tumor growth and resistance to treatment, but the specific mechanisms and mediators are not fully understood. Regulation of the CSC state is considered an ideal therapeutic strategy both in the early stages of tumor formation and within established tumors. Exosomes have emerged as key players in intercellular communication, similar to classical hormone signaling, and are essential for facilitating communication between cells in liver cancer. Here, by coupling immunomagnetic bead sorting and exosomal sequencing, we found that exosome-derived circRNAs enriched in liver cancer CSCs were the key subsets with stemness characteristics and ultimately promoted HCC development. Of interest, we found that circ-ZEB1 and circ-AFAP1 are strongly correlated with liver cancer stemness and a poor prognosis, and can regulate the epithelial-mesenchymal transition (EMT) process. Our novel exosome-derived circRNAs play a vital role as key components of various intercellular crosstalk and communication systems in malignant transmission. This finding not only provides valuable support for utilizing plasma exosomal circRNAs as clinical prognostic indicators for HCC patients but also highlights a new research direction in exploring the signaling between liver CSCs and the messenger molecules contained within exosomes.

**Supplementary Information:**

The online version contains supplementary material available at 10.1186/s12943-023-01891-y.

Hepatocellular carcinoma (HCC) remains a global challenge, with the latest GLOBOCAN 2020 data showing that HCC is the sixth most common cancer worldwide and the third leading cause of cancer-related deaths worldwide [1]. By 2025, it is estimated that more than one million people will develop liver cancer each year. Despite the many advances in the treatment of HCC in recent years, its heterogeneity, susceptibility to recurrence and metastasis and treatment resistance have made it difficult to substantially improve the five-year survival rate of patients, even in the current era of precision medicine [2, 3]. Therefore, it is crucial to explore the molecular mechanism of HCC development and explore new therapeutic targets and prognostic markers to prevent HCC progression.

Cancer stem cells (CSCs), also known as tumor-initiating cells (TICs), are an important factor in tumor development and constitute a small population of cells within tumor tissues that are capable of self-renewal and multidirectional differentiation and have strong tumorigenic capacity. The first leukemia stem cells were identified in 1997, and since then, cancer stem cells have been isolated and identified from a variety of solid tumors and tumor cell lines, including lung, prostate and pancreatic cancers [4, 5]. Studies have shown that at the early stage of tumorigenesis, tumor cells with strong stemness may have already entered the blood or lymphatic fluid and metastasized to other sites to enter a dormant state, and changes in the body's environment may promote the recovery of dormant CSCs and multidirectional differentiation, leading to tumor recurrence and metastasis. In addition, CSCs can enhance the immune system and facilitate immune escape mechanisms (e.g., through the low expression of T-cell activation markers and costimulatory molecules and increased expression of T-cell suppressor molecules such as PD-L1), and various immunotherapies targeting CSCs are currently in clinical development [6]. In HCC, cancer stem cells also play an essential role. The study of the stemness transformation of HCC cells and the role of CSCs in the development of HCC is a major focus of research in the field of HCC. In addition to their robust self-renewal and multidirectional differentiation potential, how they regulate non-CSCs in the tumor microenvironment and influence tumor development is still unclear.

Exosomes are nanoscale extracellular vesicles with a diameter of approximately 30–100 nm and are secreted by cells; their contents include DNA, RNA, lipids, and proteins, which can reflect cell metabolism, oncogenesis, and apoptosis to some extent [7, 8]. These vesicles can be delivered from donors to recipient cells, play a key role in intercellular communication, and regulate the tumor microenvironment. The interaction between cancer cells and their microenvironment plays an important role in the progression of the disease. The delivery of oncogenes to normal cells by exosomes has been demonstrated to be a mechanism of tumor invasion and metastasis under pathological conditions. The pro-tumorigenic role of exosomes has now been reported in several tumor species [9, 10]. CSCs act as major drivers of tumor development, recurrence, and metastasis, and it has been shown that CSC-derived exosomes mediate chemoresistance in pancreatic cancer cells by transferring oncogenic microRNAs [11]. The derived exosomes carry pluripotent transcription factors, such as Nanog, Oct-4, and Wnt family proteins, that can promote self-renewal and stemness transformation of recipient cells through alteration [12, 13]. In HCC, it is unclear how exosomes derive from the CSC cell population regulate other tumor cells and influence tumor development.

CircRNAs are a class of endogenous noncoding RNAs (ncRNAs) that regulate gene expression in numerous biological organisms. They have no 5' cap and no 3' poly(A) tail, and their downstream 3' splice donors are reverse-linked to the upstream 5' splice acceptors by a covalent bond, forming a loop structure [14]. Because circRNA is less susceptible to degradation by nucleases, it is more stable than linear RNA, which gives circRNA a clear advantage in terms of developing novel clinical diagnostic markers. While exosomal lncRNAs and miRNAs have been well studied, there are still many gaps in our understanding of the association between circRNAs and tumor exosomes.

The vital role of exosomes as information carriers in the stemness regulation of liver CSCs has yet to be studied. Whether they are involved in the interconversion between non-CSCs and CSCs and maintain the dynamic balance of liver CSCs and their mechanism of action, as well as whether they can target the key circRNA molecules and their signaling pathways in exosomes to remove liver CSCs and fundamentally inhibit the recurrence and metastasis of HCC, deserves further study. This study was designed to address the above mentioned research gap; hepatoma stem cells were enriched from the human hepatoma cell lines HepG2 and Huh7, exosomes were identified and isolated from hepatoma stem cell-enriched samples, and the regulatory role of hepatoma stem cell exosomes on the malignant biological behavior of hepatoma was explored. We also screened and validated the key circRNA molecules that play a regulatory role to further explore their functions and potential mechanisms.

## Results and discussion

### Liver CSC exosomes promote the malignant biological behavior of liver cancer cells

**Fig. 1:**
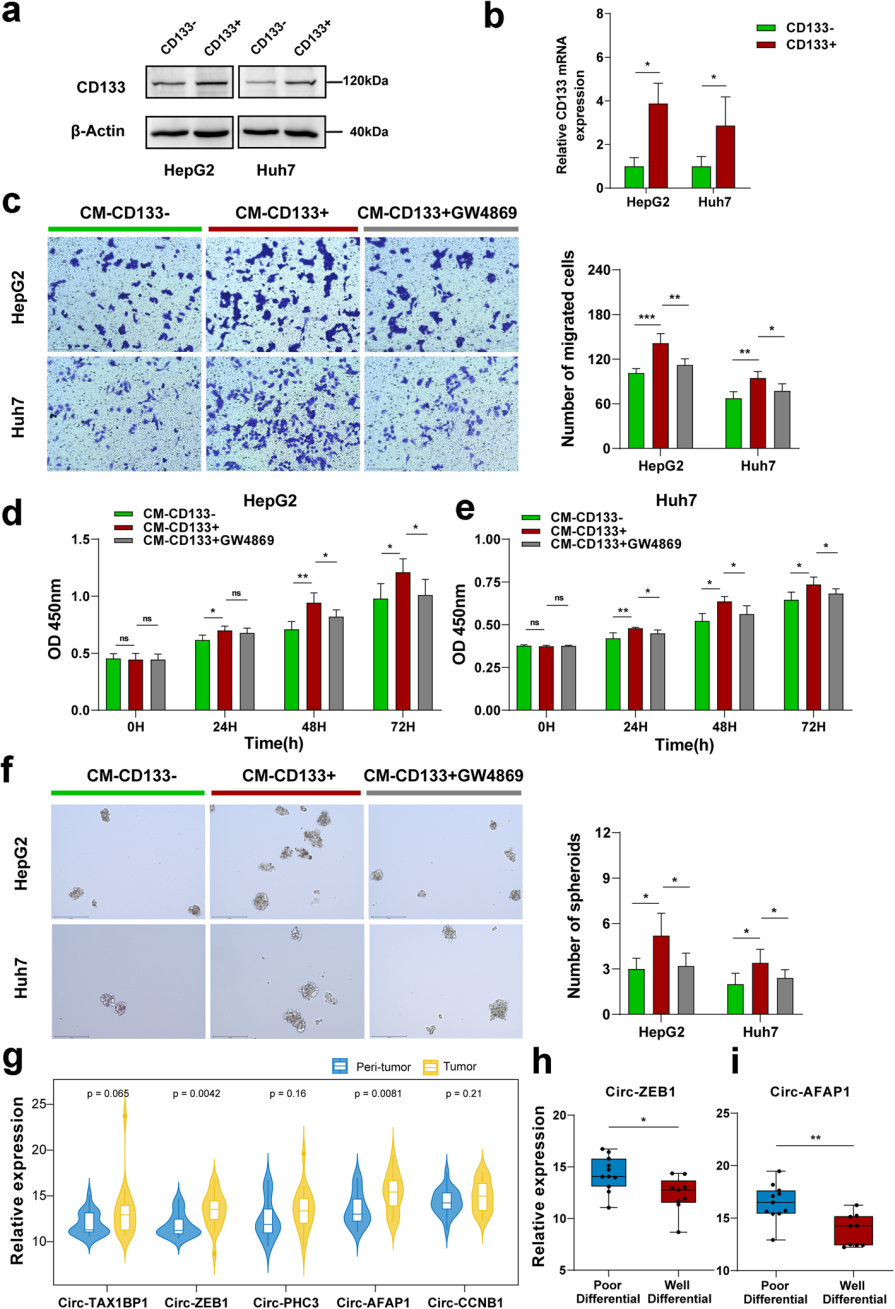
CircRNAs derived from the exosomes of liver CSCs play a significant role in malignant dissemination. **a** and **b**. Western blot and Real-time PCR analysis of the expression of CD133 in CD133 positive (CD133 +) and CD133 negative (CD133-) cells. **c**. Transwell migration assay to assess the migration ability of HepG2 and Huh7 cells cultured with conditioned medium derived from CD133- cells, CD133 + cells, and CD133 + cells treated with GW4869. **d** and **e**. Cell viability of HepG2 and Huh7 cells cultured with conditioned medium derived from CD133- cells, CD133 + cells, and CD133 + cells treated with GW4869 using the CCK-8 assay at 0 h, 24 h, 48 h, and 72 h. **f**. Spheroid formation assay to evaluate the spheroid-formation ability of HepG2 and Huh7 cells treated with conditioned medium derived from CD133- cells, CD133 + cells, and CD133 + cells treated with GW4869. **g**. Expression levels of the top 5 differentially expressed circRNAs in 20 clinical samples of liver cancer tissues and matched paraneoplastic tissues. **h** and **i**. Expression levels of circ-ZEB1 and circ-AFAP1 in well differential and poor differential liver cancer tissues. ns: not statistically significant, *P < 0.05, **P < 0.01, ***P < 0.001

To obtain liver CSCs, we isolated CD133 positive (CD133 +) HCC cells from HCC HepG2 and Huh7 cell lines using immunomagnetic beads as the experimental group and CD133 negative (CD133-) HCC cells as the control cells (Supplementary Fig. S1a) and used exosome-free serum medium for culture. It was demonstrated that liver CSCs were well enriched by the magnetic sorting method, which laid the foundation for further experiments (Fig. 1a, b).

Conditioned medium derived from CD133 + liver CSCs was used to treat cells in the experimental group, and conditioned medium derived from CD133- HCC cells were used to treat cells in the control group. Compared with that of the control group, the proliferation ability of the HepG2 experimental group was significantly increased (*p* < 0.05) (Supplementary Fig. S1b) at 48 h and 72 h. Moreover, the Huh7 cell line experimental group showed a significant increase in cell proliferation capacity at 24 h, 48 h and 72 h compared to that in the control group (*P* < 0.05) (Supplementary Fig. S1b); the HepG2 and Huh7 cell line experimental groups showed significant increases in the number of migrated cells and migration capacity when compared to the control group (*P* < 0.05) (Supplementary Fig. S1c and d); moreover, the cell spheroid -formation ability was significantly enhanced in the experimental group compared to the control group (Supplementary Fig. S1e and f). The results suggest that the conditioned medium of the liver CSC population can significantly enhance the proliferation, migration, and spheroid formation of tumor cells.

In terms of malignant biological behaviors such as proliferation, migration and stemness (reflected by spheriod formation ability) of the cells, the above results indicated significance between the experimental and control groups. Further investigation is necessary to identify the components in the conditioned medium of liver CSCs that mediate the enhanced malignant biological behaviors of liver cancer cells. Considering that tumor cell conditioned medium contains various components, including growth factors, extracellular matrix proteins, extracellular vesicles, and noncoding RNAs, some of these components can promote the malignant biological behavior of tumor cells. Extracellular vesicles (EVs) are highly potent elements found within the conditioned medium obtained from tumor cells. These vesicles contain biological components with the ability to transmit crucial information among cells. Moreover, EVs can also serve as a stable environment to prevent the external environment from inactivating or degrading internal load molecules. Compared to other components, they are essential in promoting malignant biological behavior in tumor cells.

To further investigate whether exosomes in the conditioned medium of liver CSCs mediate the malignant biological behavior of liver cancer cells, we used the exosome inhibitor GW4869 to inhibit exosome secretion by liver CSCs. We first treated CD133 + liver CSC medium with the exosome inhibitor GW4869 for 48 h, CD133 + liver CSC medium with DMSO in the control group, and CD133- HCC cell medium with DMSO for 48 h. Then, functional assays were performed 48 h after the addition of the conditioned medium to HepG2 cells and Huh7 cells. The results of the functional study showed that the migration, proliferation, and spheroid-formation ability of HepG2 and Huh7 cells decreased in the group treated with GW4869 compared to the CD133 + liver CSC group without GW4869 (*P* < 0.05) (Fig. 1c, d, e and f). This result suggested that the addition of the exosome inhibitor GW4869 inhibits the secretion of exosomes from CD133 + liver CSCs. The promotion of malignant biological behavior of HCC cells was weakened compared with that in the control group, which suggests that CD133 + liver CSCs promote the malignant biological behavior of surrounding HCC cells to some extent through the secretion of exosomes.

Considering that HepG2 cells were derived from hepatoblastoma and have better stemness formation ability and cell secretion function than Huh7 cells [15], we further performed liver CSC exosome extraction and identification and subsequent sequencing experiments to select HepG2 cells as the target cells. The cell exosomes were extracted by the exoEasy Maxi Kit. Typical exosome-like nanoparticles with membrane structures were observed under an electron microscope (Supplementary Fig. S1g); they had diameters ranging from 50–120 nm (Supplementary Fig. S1h), high expression of the extracellular vesicle structure-related proteins CD63 and TSG101, and no expression of the endoplasmic reticulum-related protein Calnexin (Supplementary Fig. S1i). Exosomes were successfully isolated and identified based on the results described above.

### CircRNA-mediated stemness-related processes in liver CSC exosomes

Whole-transcriptome sequencing was performed on the two groups of exosomes, and differential expression analysis and Gene Ontology (GO) and Kyoto Encyclopedia of Genes and Genomes (KEGG) enrichment analysis were performed on mRNA, lncRNA and circRNA from the sequencing results. The results showed that circRNA (Supplementary Fig. S2a-c) was significantly differentially expressed between CD133 + cells and control cells compared to mRNA and lncRNA and correlated with liver CSC characteristics (Supplementary Fig. S2d). To further explore circRNAs in exosomes, five potential key circRNA molecules with significance in exosomal sequencing and functional importance were screened (Table 1). Gene Ontology (GO) enrichment analysis suggested that the above screened differentially expressed genes may be involved in the regulation of the activity of rho guanyl-nucleotide exchange factor and antiporter activity (Supplementary Fig. S2e), with cellular localization mainly in postsynaptic density and synapses (Supplementary Fig. S2e). KEGG enrichment analysis further suggested that these differentially expressed genes are involved in cell adhesion molecules and calcium-related signaling pathways (Supplementary Fig. S2e). The analyses of mRNAs and lncRNAs are shown in Supplementary Fig. S3a-e and Supplementary Fig. S3f-j.

**Table 1:** Significantly different circRNAs and their modified genes in exosomes

CircBaseID	logFC	GeneName
hsa_circ_0134036	4.300490342	TAX1BP1
hsa_circ_0004907	4.298552721	ZEB1
hsa_circ_0001359	4.089470006	PHC3
hsa_circ_0069152	3.843958127	AFAP1
hsa_circ_0001495	2.913802664	CCNB1

### Circ-ZEB1 and circ-AFAP1 are strongly associated with liver cancer prognosis

Twenty primary tumor samples and matched paraneoplastic tissues were collected, and the clinical information of the patients is shown in Table 2. RNA was extracted and reverse transcribed into cDNA, and the circRNAs screened above were validated (Fig. 1g). We found that the expression of circ-ZEB1 and circ-AFAP1 was significantly higher in HCC tissues than in paraneoplastic tissues, and their expression levels were positively correlated with the expression of CD133, a stem cell marker (Supplementary Fig. S4a, b). In poor differential HCC tissues, these two molecules were expressed at significantly higher levels than in well differential HCC tissues, as shown in Fig. 1h and i. The results suggest that circ-ZEB1 and circ-AFAP1 are prognostic markers for HCC, and these findings are expected to provide a theoretical basis for further screening of potential prognostic markers.

**Table 2:** Clinicopathological characteristics of 20 HCC patients

	**Patients, n**	**Proportion, %**
**Sex**		
Male	14	70.0
Female	6	30.0
**Age**		
< 60	14	70.0
≥ 60	6	30.0
**Pathological differentiation**		
Poor differentiation	11	55.0
Well differentiation	9	45.0
**Tumor diameter**		
≤ 5cm	8	40.0
> 5cm	12	60.0
**Tumor capsule**		
Yes	12	60.0
No	8	40.0
**Macrovascular invasion**		
Yes	3	15.0
No	17	85.0
**Tumor number**		
Single	16	80.0
Multiple	4	20.0
**Cirrhosis**		
Yes	11	55.0
No	9	45.0
**AFP (ng/ml)**		
≤ 400	13	65.0
> 400	7	35.0
**Hepatitis B virus infection**		
Yes	6	30.0
No	14	70.0

**Fig. 2:**
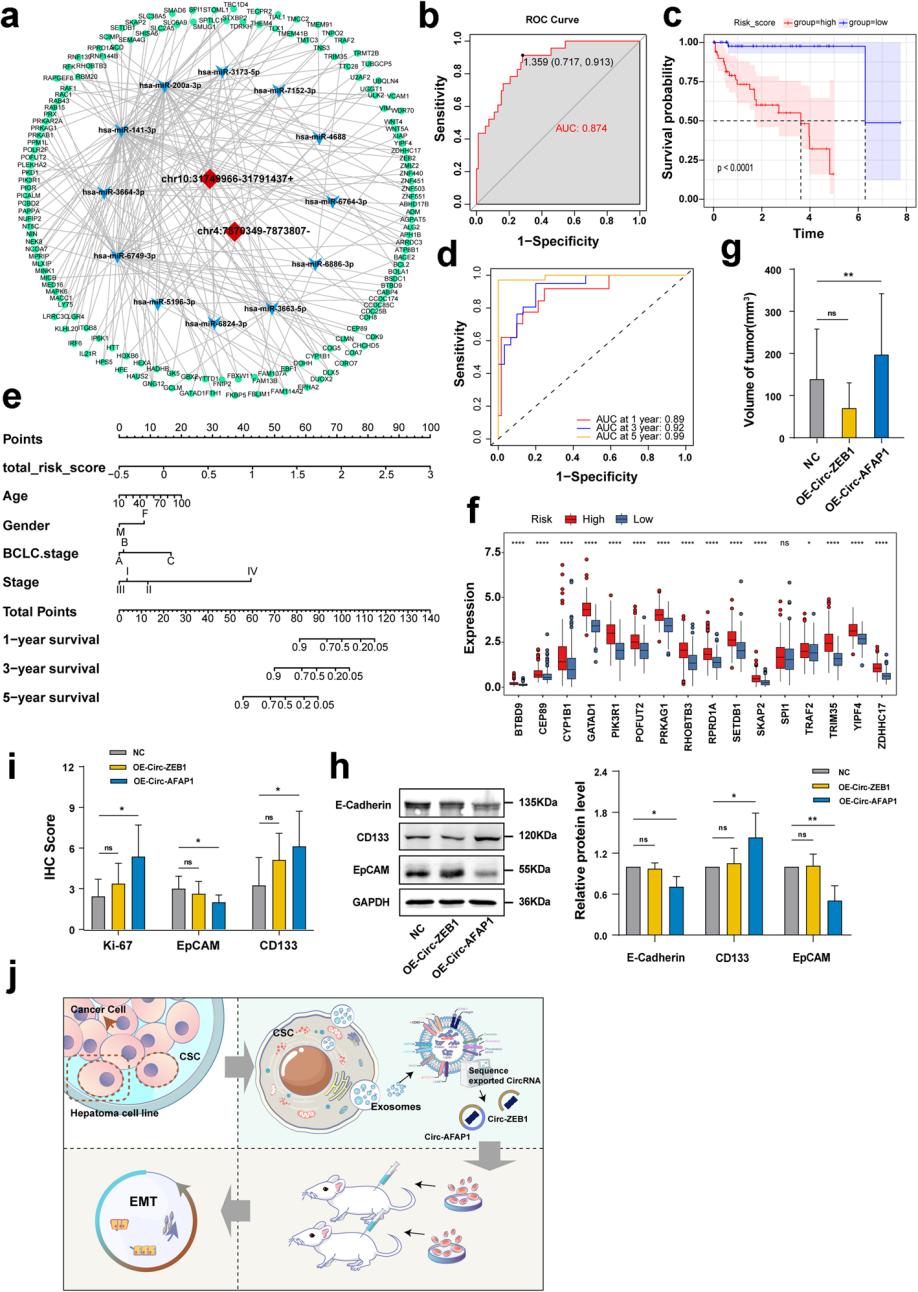
Circ-ZEB1 and circ-AFAP1 are closely correlated with poor prognosis and progression of liver cancer. **a**. CeRNA network consisting of two circRNAs (central red nodes), miRNAs (inner blue nodes) and mRNAs (outer green nodes). **b**. ROC curve for model evaluation. **c**. Kaplan‒Meier survival curves for patients in high-risk and low-risk groups based on the mRNA risk model. **d**. Time-dependent ROC curve for the risk model at 1-year, 3-year, and 5-year intervals. **e**. Nomogram based on the 16 mRNAs in the risk model. **f**. Expression profiles of genes in the high-risk and low-risk groups of the TCGA database within the model. **g**. Comparison of tumor volume with and without overexpression of circ-ZEB1 and circ-AFAP1. **h**. Expression of E-cadherin, CD133, and EpCAM in tumor tissues after overexpression of circ-ZEB1 and circ-AFAP1, as detected by Western blot analysis. **i**. Immunohistochemical staining results for Ki-67, EpCAM, and CD133 protein levels in tumor tissues after circ-ZEB1 and circ-AFAP1 overexpression. **j**. Study profile. Influence of relevant circRNAs in liver CSC exosomes on the malignant biological behavior of liver cancer. ns: not statistically significant, *P < 0.05, **P < 0.01

To further investigate the association of circ-ZEB1 and circ-AFAP1 expression with the clinical features of HCC, we relied on the above exosomal sequencing results and public databases to construct ceRNA and risk prediction models to perform correlation analysis. The intersection of 1751 differentially expressed mRNAs from the sequencing results yielded 146 mRNAs (Supplementary Fig. S4c). Based on the above results, a ceRNA network which contained 160 nodes and 211 reciprocal relationship pairs was constructed (Fig. 2a).

Enrichment of GO and KEGG functions was performed on mRNAs in the ceRNA network. In this study, we focused on stem cell-related biological processes and pathways. Supplementary Fig. S4d shows that GO functions in two significantly different biological processes, somatic stem cell population maintenance and stem cell development, and three closely related KEGG pathways, the Wnt signaling pathway, PI3K-Akt signaling pathway and MAPK signaling pathway (Supplementary Fig. S4e).

A risk assessment model (GSE76427) was constructed to assess the relevance of mRNAs in the ceRNA network to the clinical characteristics of HCC patients based on LASSO regression with an AUC value of 0.874 (Fig. 2b, Supplementary Fig. S4f and g). Moreover, the genes with the most potent predictive power were identified using a multifactor Cox regression model, and a total of 16 genes were identified. The result is presented as a forest plot (Supplementary Fig. S4h). These 16 genes were subsequently used to calculate the risk score for patients with liver cancer as follows: risk score = (0.0023 × BTBD9 expression) + (-0.0036 × CEP89 expression) + (0.0001 × CYP1B1 expression) + (0.0027 × GATAD1 expression) + (0.0002 × PIK3R1 expression) + (0.0003 × POFUT2 expression) + (0.0006 × PRKAG1 expression) + (-0.0008 × RHOBTB3 expression) + (0.0005 × RPRD1A expression) + (-0.0012 × SETDB1 expression) + (-0.0007 × SKAP2 expression) + (-0.0007 × SPI1 expression) + (-0.0023 × TRAF2 expression) + (0.0054 × TRIM35 expression) + (0.0011 × YIPF4 expression) + (0.0035 × ZDHHC17 expression). Patients were divided into high-risk and low-risk groups based on the median risk score. The five-year survival rate was significantly lower in the high-risk group than in the low-risk group (Fig. 2c). TimeROC analysis showed that the model predictions had AUC values of 0.89, 0.92 and 0.99 for 1-year, 3-year and 5-year survival in liver cancer patients, respectively (Fig. 2d). According to the nomogram and calibration curve, the risk model was found to be a better predictor of 5-year survival (Fig. 2e and Supplementary Fig. S5f). To further assess the relationship between survival and the risk score and clinicopathological characteristics of the patients, univariate and multifactor Cox analyses were performed. The results of the univariate Cox analysis showed that age and gender were not associated with overall survival (Supplementary Fig. S5a). However, BCLC stage and risk score were significantly associated with overall survival, and the results of the multifactor Cox analysis showed that only risk score was significantly associated with overall survival (Supplementary Fig. S5b).

Based on the risk score formula of the above model, TCGA liver cancer patients were divided into high and low groups according to the median risk score. According to the results, the model genes (except for the SPI1 gene) were significantly highly expressed in the high-risk group. The expression of the SPI1 gene was not significantly different between the two groups, although this gene showed a trend of high expression in the high-risk group (Fig. 2f).

Targeted therapy for hepatocellular carcinoma is being developed rapidly, and molecular targeted therapy has now become a milestone in the clinical disease management of patients with intermediate to advanced hepatocellular carcinoma. In recent years, immunotherapy combined with targeted therapy has emerged as a breakthrough combination and has shown promising initial results. The protein crystal structures of the core genes CYP1B1, PIK3R1, POFUT2, PRKAG1, SETDB1, SKAP2, TRAF2 and ZDHHC17 in the mRNA prediction model were molecularly docked with the commonly used hepatocellular carcinoma targeting drugs Cabozantinib, Donafenib, Lenvatinib, Regorafenib and Sorafenib in clinical practice (BTBD9, CEP89, GATAD1, RHOBTB3, RPRD1A, SPI1, TRIM35, YIPF4 crystal structures were not found). The lowest binding energy (< -5.0 kcal/mol) is generally considered to indicate good binding activity between the active molecule and the protein, and a lower binding energy indicates a stronger interaction between the molecule and the protein. The docking results showed that the binding energies were < -5.0 kcal/mol (Supplementary Fig. S5c), indicating that the core targets in this model may have good binding efficacy for common targeted drugs.

### Overexpression of circ-AFAP1 promotes tumor growth and enhances liver cancer cell stemness and EMT

The tumorigenic capacity of BALB/c nude mice is a crucial indicator of the malignant characteristics of tumor cells. To evaluate the effect of circ-ZEB1 and circ-AFAP1 on tumor growth in vivo, JHH-7 cells transfected with OE-circ-ZEB1, OE-circ-AFAP1 and negative control virus CON254 were inoculated subcutaneously on the upper backs of 5-week-old BALB/c nude mice. Tumor volume was first measured at Day 5 and recorded every 2 days. Seven days later, the mice were sacrificed, and the tumors were collected (Supplementary Fig. S5d). Tumor volume and weight were then measured. The tumors were removed, fixed in paraformaldehyde, embedded in paraffin wax, and further sectioned for immunohistochemistry to detect stemness-related parameters. The results showed no significant change in weight in the three groups of mice (Supplementary Fig. S5e), while the tumor volume was significantly higher in the circ-AFAP1 overexpression group than in the control group (Fig. 2g and Supplementary Fig. S5g). The circ-ZEB1 overexpression group showed no significance in tumor volume and weight compared to the control group (Fig. 2g and Supplementary Fig. S5g). After extracting tumor tissue proteins from mice and detecting stemness and EMT-related molecules, it was found that the expression of CD133 was significantly increased in the OE-circ-AFAP1 group in comparison with the NC group. E-cadherin and EpCAM were downregulated (*P* < 0.05) (Fig. 2h). The results of immunohistochemistry experiments on Ki67, CD133 and EpCAM showed that (Supplementary Fig. S6) the Ki67 and CD133 scores of tumor cells in the OE-circ-AFAP1 group were higher than those in the control group, the EpCAM scores showed no significance compared with the control group (Fig. 2i), and no significance was observed in the OE-circ-ZEB1 group compared with the control group. Circ-AFAP1 overexpression may promote HCC progression by enhancing tumor cell stemness and the EMT process.

To definitively confirm the origin of these two clinically relevant circRNAs in other types of HCC stem cells, we employed immunomagnetic beads to isolate cells expressing the positive markers for HCC stem cells, including cell surface vimentin (CSV) and CD90. In conjunction with immunofluorescence and real-time PCR results, our findings demonstrated successful isolation of CSV positive stem cells and CD90 positive stem cells (Supplementary Fig. S7a, b and c). The real-time PCR data revealed a significant upregulation in the expression of circ-AFAP1 in CSV positive and CD90 positive stem cells when compared to their CSV negative and CD90 negative counterparts (*p* < 0.05) (Supplementary Fig. S7b, c, d, and e). However, no significant differences were observed in the expression of circ-ZEB1 (*p* > 0.05) between these two cell groups (Supplementary Fig. S7b, c, d, and e).

Our preliminary experiments (Fig. 2j) confirmed that some key circRNAs play an essential role in exosomes secreted by liver CSCs as messenger molecules with more stable traits than other messenger molecules, so circRNAs may mediate the regulatory effects of CSCs on non-CSCs. Furthermore, circRNAs may promote HCC progression by enhancing tumor cell stemness and EMT processes. The discovery of the above circRNAs in the exosomes of liver cancer patients and detection of their expression levels as indicators of poor prognosis will be of substantial clinical relevance and significance if our experiment can be further validated.

## Conclusion

Cancer stem cells and exosomes play an important role in the development of hepatocellular carcinoma. Our study revealed that circ-ZEB1 and circ-AFAP1, derived from liver CSC exosomes, may play an essential role in the crosstalk between CSCs and non-CSCs. They may mediate the enhanced malignant biological behavior of non-CSCs and poor prognosis of HCC patients by enhancing the stemness of liver cancer cells and the EMT process. This study initially revealed that key circRNAs in exosomes of liver cancer CSCs can act as key mediators of malignant transmission, which is expected to provide theoretical support for the clinical translation of plasma exosomal key circRNAs in HCC patient prognosis prediction, and provide a new research direction for studying regulation relationships and signaling between liver CSCs and messenger molecules in exosomes.

### Supplementary Information

**Additional file 1: Supplementary Figure S1.** Exosomes derived from CD133+ cells enhance the malignant biological behavior of liver cancer cells. a. Morphology of CD133+ and CD133- cells isolated by immunomagnetic beads. b. Cell viability of HepG2 and Huh7 cells cultured with conditioned medium derived from CD133+ and CD133- cells from HepG2 and Huh7 cells using the CCK-8 assay at 0 h, 24 h, 48 h and 72 h. c and d. Transwell migration assay to assess the migration ability of HepG2 and Huh7 cells cultured with conditioned medium derived from CD133+ and CD133- cells at 48 h. e and f. Spheroid formation assay to evaluate the spheroid-formation ability of HepG2 and Huh7 cells treated with conditioned medium derived from CD133+ and CD133- cells. g. Transmission electron microscopy (TEM) image of exosomes extracted from the cell culture conditioned medium showing a typical saucer-shaped morphology. h. Nanoparticle Tracking Analysis (NTA) particle size analysis indicated a diameter ranging from 50-120 nm. i. Western blot analysis of the exosome markers CD63 and TSG101, as well as the endoplasmic reticulum marker calnexin, in exosomes derived from CD133+ CSC and CD133- cell culture conditioned medium. ns: not statistically significant, *P<0.05, **P<0.01, ***P<0.001.

**Additional file 2: Supplementary Figure S2.** CircRNAs identified in exosomal sequencing results are closely related to cellular stemness. a, b and c. Heatmap, scatter plot, and volcano plot of differentially expressed circRNAs identified in exosomal sequencing results. d. GO analysis and KEGG pathway enrichment analysis of differentially expressed circRNAs showing stem cell-related biological processes and pathways. e. Top 10 significantly enriched molecular functions, cell components and KEGG pathways of identified differentially expressed circRNAs based on GO analysis.

**Additional file 3: Supplementary Figure S3.** Bioinformatics analysis of mRNAs and lncRNAs in exosomal sequencing results. a, b and c. Differential analysis, GO and KEGG enrichment analysis were applied to identify the biological processes and pathways related to stemness. Heatmaps, scatter plots and volcano plots were created for differential mRNA expression analysis. d and e. GO and KEGG enrichment analysis results of stemness-related biological processes and pathways for mRNA are displayed. f, g and h. Heatmaps, scatter plots and volcano plots were created for differential lncRNA expression analysis. i and j. GO and KEGG enrichment analysis results of dryness-related biological processes and pathways for lncRNAs are displayed.

**Additional file 4: Supplementary Figure S4.** Construction of the ceRNA network and mRNA risk model. a and b. Correlation of the expression levels of circ-ZEB1, circ-AFAP1 and CD133 in liver cancer tissues. c. Venn diagram showing the intersection between miRNA target genes and mRNAs identified in exosomal sequencing. d and e. GO and KEGG pathway enrichment of the mRNAs in the ceRNA network, showing stem cell-related biological processes and pathways. f and g. LASSO regression analysis to construct a prognostic risk model and ROC curve for model evaluation. h. Forest plot of multivariate Cox analysis showing the 16 mRNAs selected as predictors in the optimal prognostic model.

**Additional file 5: Supplementary Figure S5.** Cox regression analysis, molecular docking and effect of circ-ZEB1 and circ-AFAP1 overexpression in vivo tumorigenicity. a and b. Univariate and multivariate Cox regression analyses of clinical features and risk score. c. Molecular docking results of genes in the risk model with commonly used targeted drugs in HCC. d. A subcutaneous tumorigenesis assay was conducted to assess how the overexpression of circ-ZEB1 and circ-AFAP1 influences the in vivo tumorigenicity of liver cancer cells in a nude mouse model. e. Changes in body weight of nude mice during the experiment. f. Calibration curve based on the 16 mRNAs in the risk model. g. Comparison of tumor volume with and without intervention of circ-ZEB1 and circ-AFAP1. ns: not statistically significant, *P<0.05, **P<0.01.

**Additional file 6: Supplementary Figure S6.** Immunohistochemical staining results in nude mouse tumor tissues. a. H&E staining showing cell morphology in nude mouse tumor tissues after intervention with circ-ZEB1 and circ-AFAP1. b. Immunohistochemical staining for CD133 protein levels in nude mouse tumor tissues after circ-ZEB1 and circ-AFAP1 intervention. c. Immunohistochemical staining for Ki-67 protein levels in nude mouse tumor tissues after circ-ZEB1 and circ-AFAP1 intervention. d. Immunohistochemical staining for EpCAM protein levels in nude mouse tumor tissues after circ-ZEB1 and circ-AFAP1 intervention. Scale indicates 50 μm.

**Additional file 7: Supplementary Figure S7.** Investigating the expression of circ-ZEB1 and circ-AFAP1 in CSV positive and CD90 positive liver cancer cells. a. Immunofluorescence showed CSV positive staining after immunomagnetic bead sorting in Huh7 and HepG2 cells. b and c. Real-time PCR was performed to explore the expression of CD90, circ-ZEB1, and circ-AFAP1 in CD90 positive and CD90 negative cells of Huh7 and HepG2. d and e. Real-time PCR was utilized to examine the expression of circ-ZEB1 and circ-AFAP1 in CSV positive and CSV negative cells of Huh7 and HepG2. Scale indicates 50 μm.

**Additional file 8. **Materials and methods.

## Data Availability

The data used and/or analyzed in the current study are available from the corresponding authors on request.

## Abbreviations

CSCs: Cancer stem cells
HCC: Hepatocellular carcinoma
EMT: Epithelial-to-mesenchymal transition
TICs: Tumor initiating cells
circRNAs: Circular RNAs
ncRNAs: Non-coding RNAs
DMEM: Dulbecco's modified eagle medium
FBS: Fetal bovine serum
Evs: Extracellular vesicles
WB: Western blot
TCGA: The Cancer Genome Atlas
GO: Gene Ontology
KEGG: Kyoto Encyclopedia of Genes and Genomes
ceRNAs: Competitive endogenous RNAs
IHC: Immunohistochemistry
HE: Hematoxylin–eosin staining
TEM: Transmission electron microscopy
NTA: Nanoparticle tracking analysis
CSV: Cell surface vimentin
