# Supplementary figures and images for: Significant CircRNAs in liver cancer stem cell exosomes: mediator of malignant propagation in liver cancer?
Source: Mol Cancer. 2023 Dec 5;22:197. doi: 10.1186/s12943-023-01891-y (PMC10696692; doi:10.1186/s12943-023-01891-y)

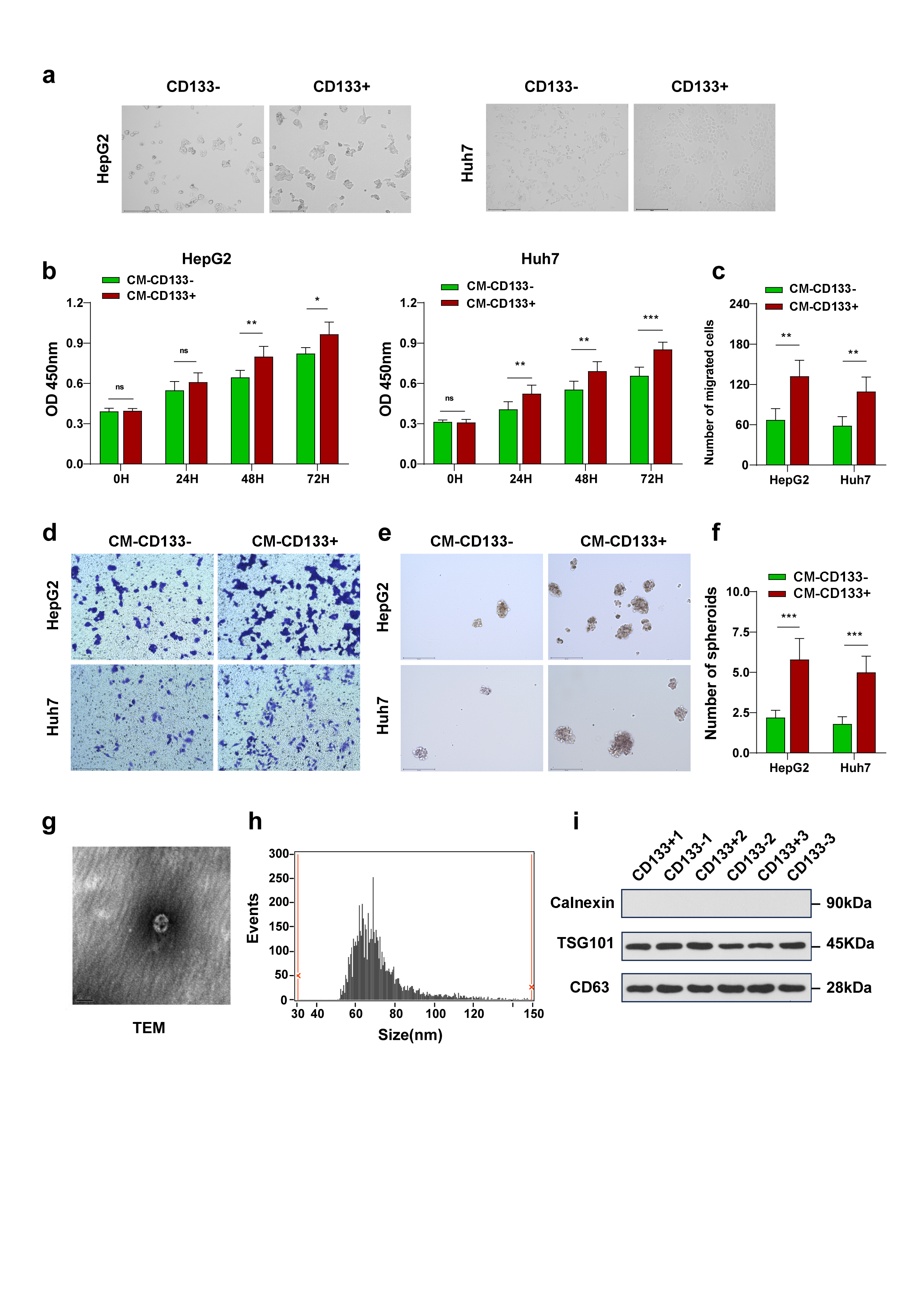

Supplement: Supplementary file 1 — Additional file 1: Supplementary Figure S1. Exosomes derived from CD133+ cells enhance the malignant biological behavior of liver cancer cells. a. Morphology of CD133+ and CD133- cells isolated by immunomagnetic beads. b. Cell viability of HepG2 and Huh7 cells cultured with conditioned medium derived from CD133+ and CD133- cells from HepG2 and Huh7 cells using the CCK-8 assay at 0 h, 24 h, 48 h and 72 h. c and d. Transwell migration assay to assess the migration ability of HepG2 and Huh7 cells cultured with conditioned medium derived from CD133+ and CD133- cells at 48 h. e and f. Spheroid formation assay to evaluate the spheroid-formation ability of HepG2 and Huh7 cells treated with conditioned medium derived from CD133+ and CD133- cells. g. Transmission electron microscopy (TEM) image of exosomes extracted from the cell culture conditioned medium showing a typical saucer-shaped morphology. h. Nanoparticle Tracking Analysis (NTA) particle size analysis indicated a diameter ranging from 50-120 nm. i. Western blot analysis of the exosome markers CD63 and TSG101, as well as the endoplasmic reticulum marker calnexin, in exosomes derived from CD133+ CSC and CD133- cell culture conditioned medium. ns: not statistically significant, *P<0.05, **P<0.01, ***P<0.001. [file 12943_2023_1891_MOESM1_ESM.tif]

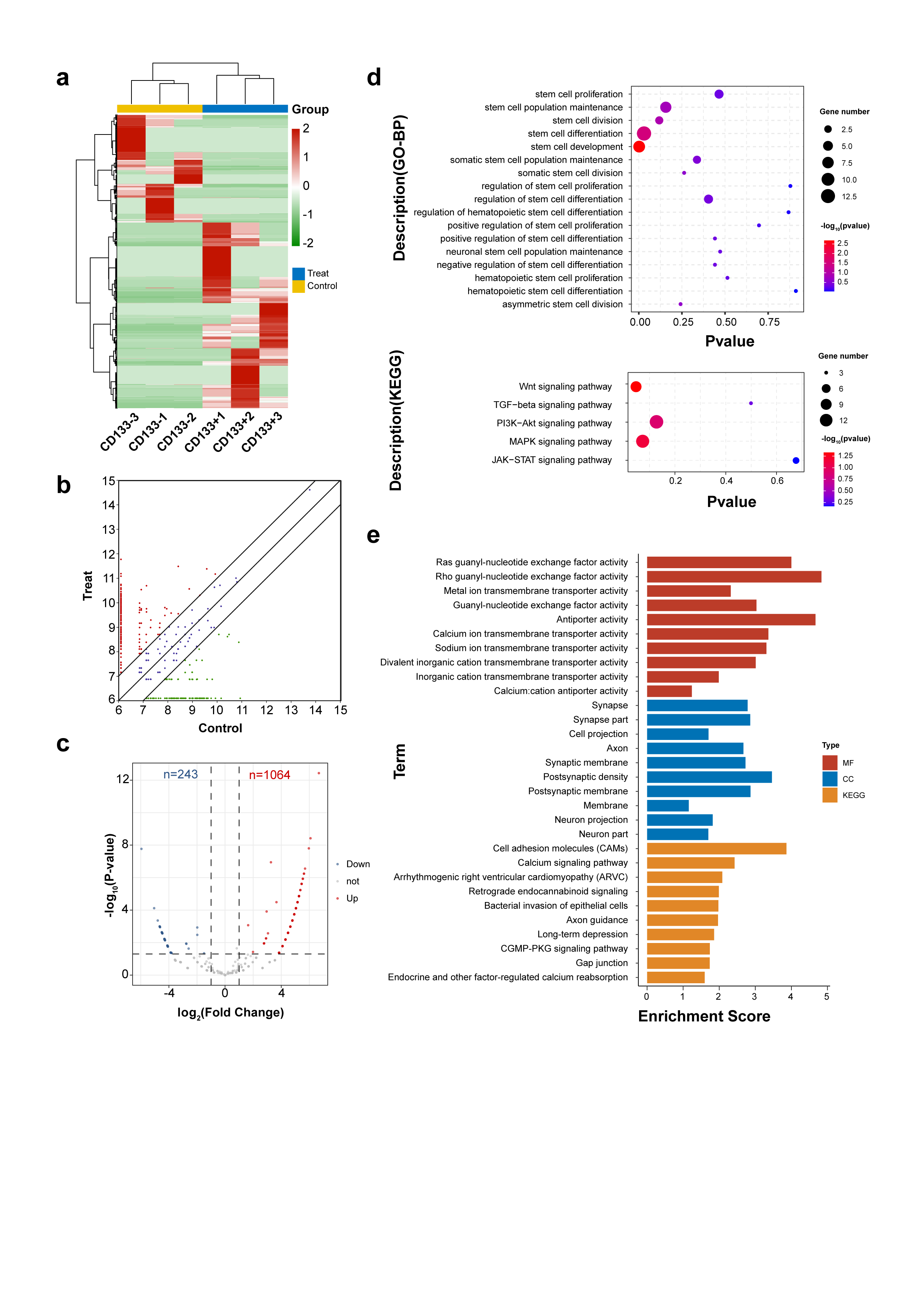

Supplement: Supplementary file 2 — Additional file 2: Supplementary Figure S2. CircRNAs identified in exosomal sequencing results are closely related to cellular stemness. a, b and c. Heatmap, scatter plot, and volcano plot of differentially expressed circRNAs identified in exosomal sequencing results. d. GO analysis and KEGG pathway enrichment analysis of differentially expressed circRNAs showing stem cell-related biological processes and pathways. e. Top 10 significantly enriched molecular functions, cell components and KEGG pathways of identified differentially expressed circRNAs based on GO analysis. [file 12943_2023_1891_MOESM2_ESM.tif]

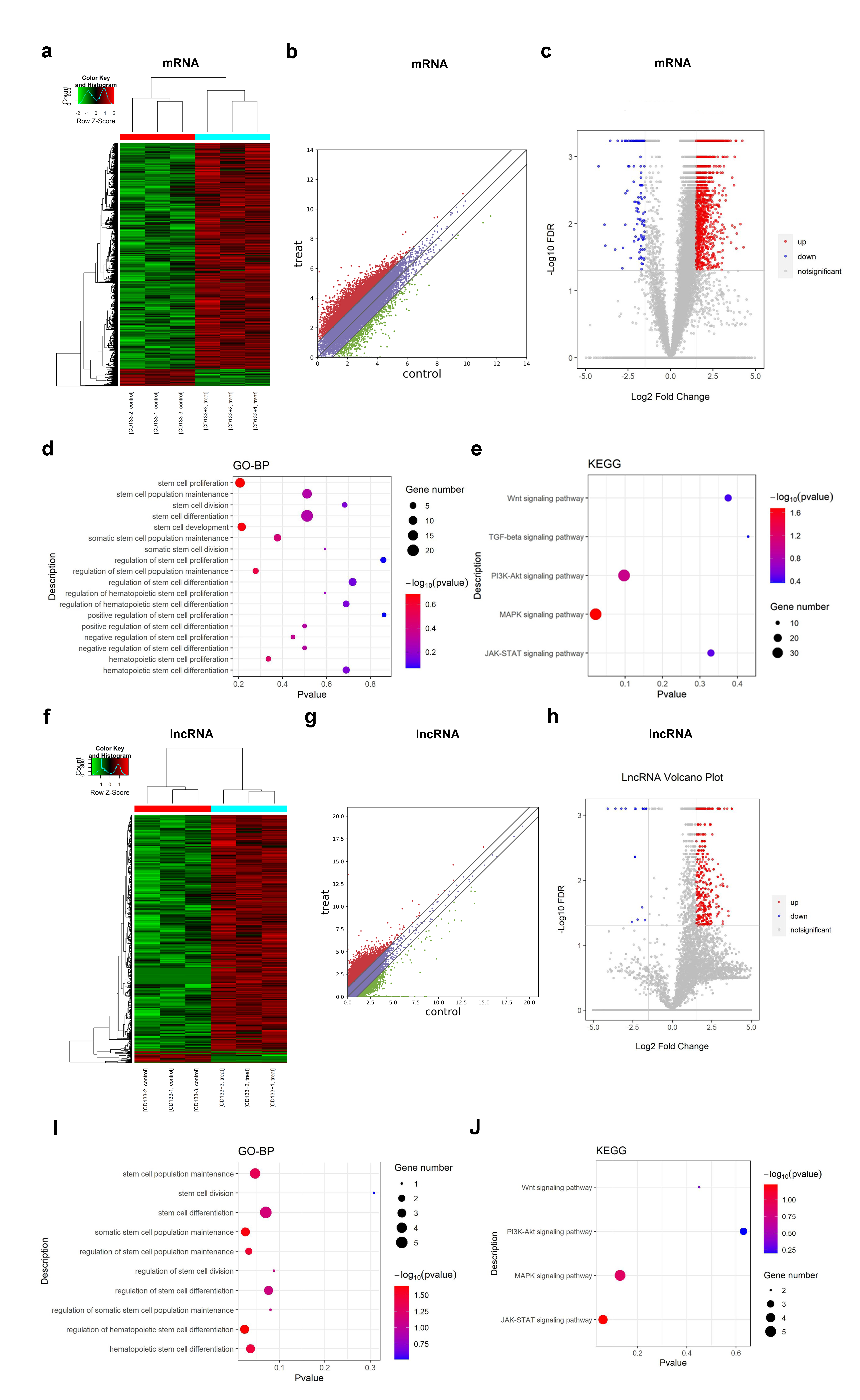

Supplement: Supplementary file 3 — Additional file 3: Supplementary Figure S3. Bioinformatics analysis of mRNAs and lncRNAs in exosomal sequencing results. a, b and c. Differential analysis, GO and KEGG enrichment analysis were applied to identify the biological processes and pathways related to stemness. Heatmaps, scatter plots and volcano plots were created for differential mRNA expression analysis. d and e. GO and KEGG enrichment analysis results of stemness-related biological processes and pathways for mRNA are displayed. f, g and h. Heatmaps, scatter plots and volcano plots were created for differential lncRNA expression analysis. i and j. GO and KEGG enrichment analysis results of dryness-related biological processes and pathways for lncRNAs are displayed. [file 12943_2023_1891_MOESM3_ESM.tif]

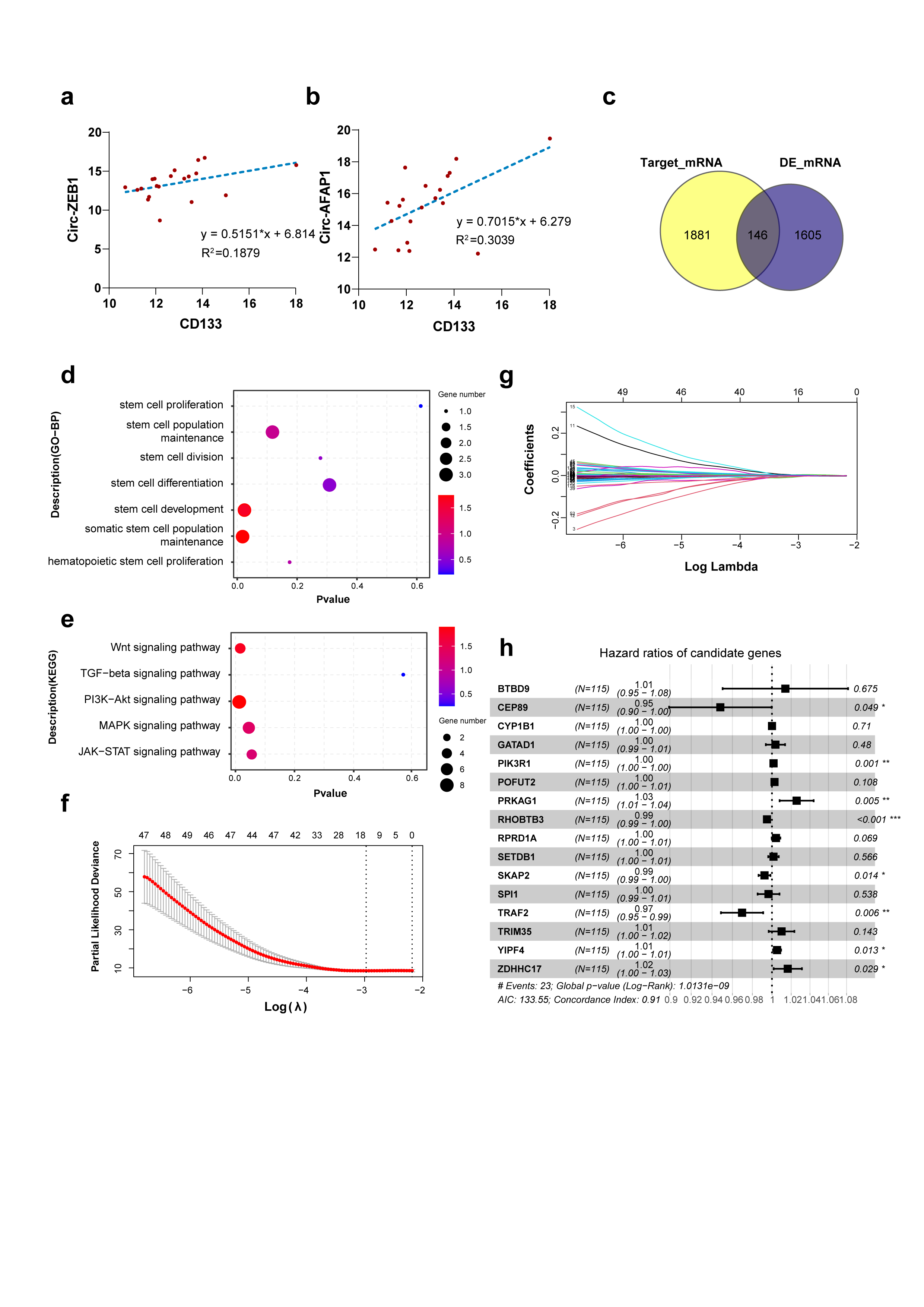

Supplement: Supplementary file 4 — Additional file 4: Supplementary Figure S4. Construction of the ceRNA network and mRNA risk model. a and b. Correlation of the expression levels of circ-ZEB1, circ-AFAP1 and CD133 in liver cancer tissues. c. Venn diagram showing the intersection between miRNA target genes and mRNAs identified in exosomal sequencing. d and e. GO and KEGG pathway enrichment of the mRNAs in the ceRNA network, showing stem cell-related biological processes and pathways. f and g. LASSO regression analysis to construct a prognostic risk model and ROC curve for model evaluation. h. Forest plot of multivariate Cox analysis showing the 16 mRNAs selected as predictors in the optimal prognostic model. [file 12943_2023_1891_MOESM4_ESM.tif]

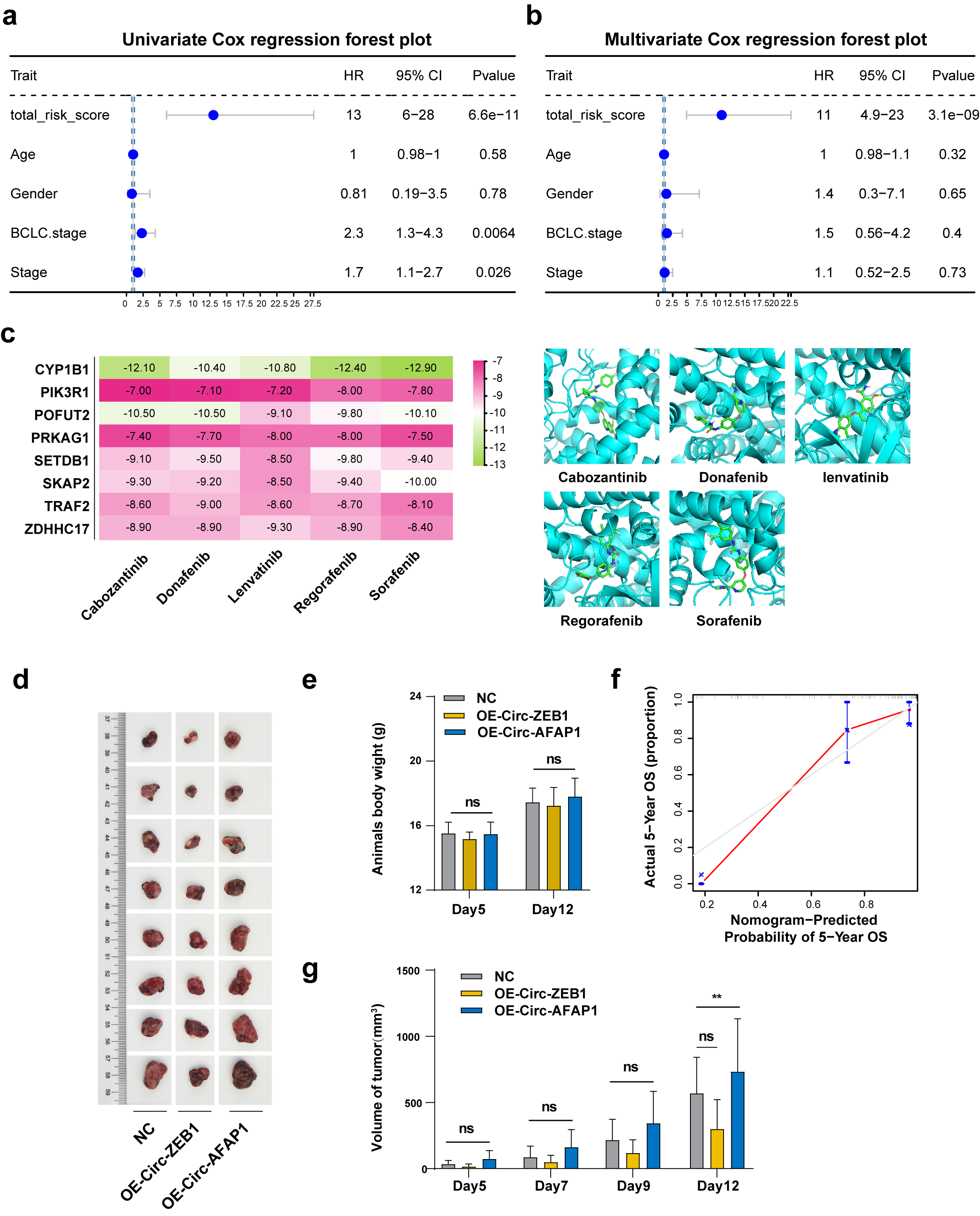

Supplement: Supplementary file 5 — Additional file 5: Supplementary Figure S5. Cox regression analysis, molecular docking and effect of circ-ZEB1 and circ-AFAP1 overexpression in vivo tumorigenicity. a and b. Univariate and multivariate Cox regression analyses of clinical features and risk score. c. Molecular docking results of genes in the risk model with commonly used targeted drugs in HCC. d. A subcutaneous tumorigenesis assay was conducted to assess how the overexpression of circ-ZEB1 and circ-AFAP1 influences the in vivo tumorigenicity of liver cancer cells in a nude mouse model. e. Changes in body weight of nude mice during the experiment. f. Calibration curve based on the 16 mRNAs in the risk model. g. Comparison of tumor volume with and without intervention of circ-ZEB1 and circ-AFAP1. ns: not statistically significant, *P<0.05, **P<0.01. [file 12943_2023_1891_MOESM5_ESM.tif]

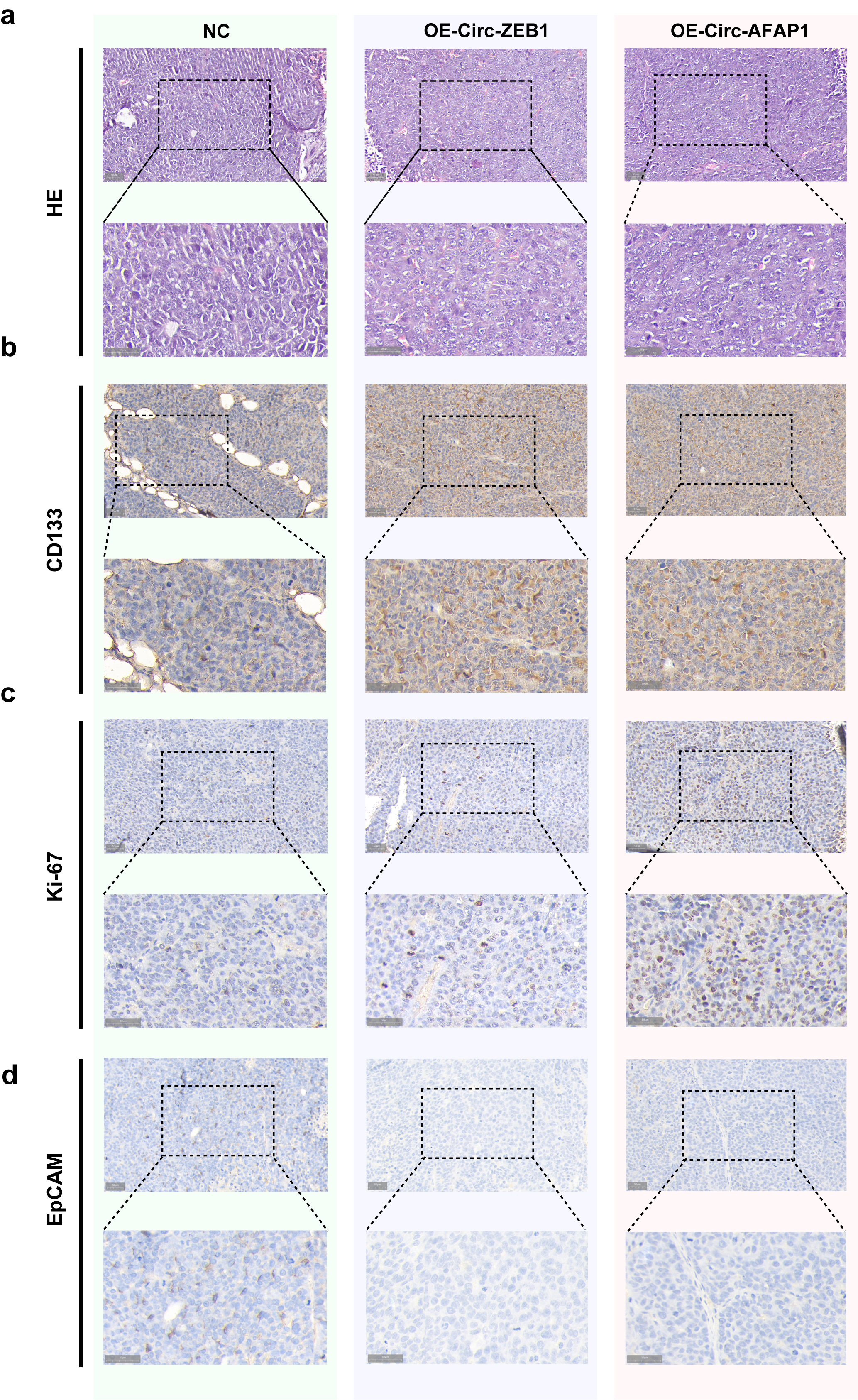

Supplement: Supplementary file 6 — Additional file 6: Supplementary Figure S6. Immunohistochemical staining results in nude mouse tumor tissues. a. H&E staining showing cell morphology in nude mouse tumor tissues after intervention with circ-ZEB1 and circ-AFAP1. b. Immunohistochemical staining for CD133 protein levels in nude mouse tumor tissues after circ-ZEB1 and circ-AFAP1 intervention. c. Immunohistochemical staining for Ki-67 protein levels in nude mouse tumor tissues after circ-ZEB1 and circ-AFAP1 intervention. d. Immunohistochemical staining for EpCAM protein levels in nude mouse tumor tissues after circ-ZEB1 and circ-AFAP1 intervention. Scale indicates 50 μm. [file 12943_2023_1891_MOESM6_ESM.tif]
