# Supplementary material for: Significant CircRNAs in liver cancer stem cell exosomes: mediator of malignant propagation in liver cancer?
Source: Mol Cancer. 2023 Dec 5;22:197. doi: 10.1186/s12943-023-01891-y (PMC10696692; doi:10.1186/s12943-023-01891-y)
Supplement: Supplementary file 8 — Additional file 8. Materials and methods. [file 12943_2023_1891_MOESM8_ESM.docx]

**Materials and methods**

**Materials**

Tissue samples from all 20 patients in this study were obtained from liver puncture or hepatectomy performed at the Seventh People’s Hospital (Shanghai, China) and General Hospital of Northern Theater Command (Shenyang, China). The samples were unanimously diagnosed as HCC by 2 senior pathologists. Detailed clinicopathological characteristics are described in Table 2. All of the above studies were approved by the Ethics Committee of the Seventh People’s Hospital of Shanghai University (2020-7th-HIRB-018) and General Hospital of Northern Theater Command (k (2017) No. 12) and conducted in accordance with the ethical guidelines of the World Medical Association Declaration of Helsinki.

**Cell lines and lentivirus**

Human HCC cell lines HepG2, Huh7 and JHH-7 cells were purchased from the Cell Resource Centre, Shanghai Academy of Life Sciences, Chinese Academy of Sciences. HCC cells were cultured in Dulbecco’s modified Eagle medium (DMEM) supplemented with 10% fetal bovine serum (FBS) and maintained in an incubator with 5% CO2 at 37°C. Infected with overexpression lentivirus LVKL84495-1 (OE-circ-ZEB1), LVKL84496-1 (OE-circ-AFAP1), and negative control virus LVCON254 (NC) are purchased from Gikai Genetics, China.

**Cell culture for exosome function validation**

Cells were cultured using DMEM complete medium containing 10% fetal bovine serum and 1% PS double antibodies in a cell incubator containing 5% CO_2_ at 37°C for 24 h. Then the supernatant of CD133+ hepatocellular carcinoma stem cells were added to the corresponding cell lines as culture medium, respectively, and the conditioned medium of CD133- hepatocellular carcinoma cell culture was added to the corresponding cell lines were marked as the control group. CCK-8 cell proliferation, Transwell cell migration assays and sphere-forming experiments were performed after the density of cells reached 70%-80% in both groups.

**Immunomagnetic bead sorting of liver cancer stem cells**

After digestion with 0.25% trypsin, the cell suspension was centrifuged at low speed (600 g for 5 min), the supernatant was removed, the cells were resuspended, and the suspension was filtered through a 30-um nylon mesh. The cell suspension obtained in the previous step was centrifuged at low speed (300 g, 10 min), and the supernatant was discarded. 300 µL per108 cells of buffer were resuspended; FcR-Blocking (100 µL per 10^8^ cells) were added and mixed well to reduce non-specific binding; CD133 Micro Beads (100 µL/10^8^ cells) were added and mixed at 4°C for 30 min. Add 2 ml of Cell Buffer/10^8^ cells to the above cell suspension, centrifuge at low speed (300 g for 10 min), discard the supernatant, then resuspend and mix gently with Cell Buffer(500 µL/10^8^ cells). The LS column was removed from the magnetic field, 1 ml of cell buffer was added, and the bead-labelled CD133+ HepG2 and Huh7 cells were collected by rapidly pushing the push plug.

**Extraction and identification of exosomes and whole transcriptome sequencing**

Follow the steps of the exoEasy Maxi Kit; the main steps are as follows: (i) Add 1 volume of binding buffer (XBP) to 1 volume of cell supernatant sample, gently invert the centrifuge tube 5 times and mix immediately and leave the mixture at room temperature; (ii) Add the sample/XBP buffer mixture to the exoEasy centrifuge column and centrifuge at 500g for 1 minute. Discard the flow-through solution and place the column in the same collection tube; (iii) Add 10 ml of Washing Buffer (XWP) and centrifuge at 3000 g for 5 minutes to remove the residual volume from the column. Discard all flow-through solution from the collection tube; (iv) Transfer the centrifuge column to a new collection tube; (v) Add 600 ul of buffer containing inorganic salts (XE) to the membrane and incubate for 1 minute. The eluate was collected by centrifugation at 500 g for 5 min; (vi) The eluate was re-added to the exoEasy centrifuge column and incubated for 1 min, centrifuged at 5000 g for 5 min, the eluate was collected and transferred to a new collection tube. The exosomes were subjected to particle size detection using a Malvern nanoparticle size analyser and combined with the expression results of CD63, TSG101 and Calnexin in Western Blot (WB) for exosome identification. The whole transcriptome sequencing of exosomes was done by Shanghai Cloud Sequence Biology Company.

**CCK8 assay**

The cells in wall culture were counted after digestion using 0.25% trypsin and media resuspension. Cell suspensions of 5000 cells/well were inoculated in 96-well plates and incubated at 37°C in a 5% CO_2_ incubator, and 10ul CCK-8 solution was added to each well of the plate after 0h, 24 and, 48h and 72h, respectively. The plates were incubated for 2 h in the incubator and the absorbance values at 450 nm were measured.

**Transwell migration**

A single cell suspension was prepared and resuspended in serum-free medium, and the cell density was adjusted to 1*10^5^ cells/ml. 200 µl of the cell suspension was added to the Transwell using a 24-well plate, and 500 µl of DMEM medium containing 20% fetal bovine serum and 1% PS double antibody was slowly added along the side wall of the lower chamber to avoid the generation of air bubbles between the chamber and the lower chamber. After 24 h incubation in a conventional incubator at 37℃ with 5% CO_2_, the Transwell was removed and washed twice with PBS, fixed with methanol at room temperature for 30 min, discarded the fixative and dried naturally, stained with 0.1% crystal violet at room temperature for 30 min, washed three times with PBS and then gently swabbed with a moistened cotton swab to remove the cells from the inner bottom of the chamber, counted and observed under an inverted microscope and photographed.

**Cell spheroid-formation assay**

The medium was configured according to 2% B27, 20ng/ml EGF, 20ng/ml bFGF and DMEM/F12, and the cells were cultured in suspension in low adhesion six-well plates with 5000 cells per well, and cell sphericity was checked and photographed after one week.

**Experimental animal models**

Logarithmically grown HCC stably transformed cells were obtained by cell passaging method using DMEM containing 10% FBS cells were resuspended in complete medium to a concentration of approximately 2 x 10^7^ cells/mL. After sterilization with alcohol, 200 μL of cell suspension was aspirated with a 1 mL syringe and injected subcutaneously into the animals. Information on the grouping of each group of BALB/c nude mice injected was recorded, and they were kept in an SPF environment after completion of injection. After measurements were completed, the BALB/c nude mice were executed by cervical dislocation and their tumour tissue was removed for immunohistochemical staining and WB. All animal procedures were performed under the approval of the Animal Experimentation Ethics Committee at the China Medical University.

**Public datasets**

mRNA chip array data of liver cancer were obtained from GEO data (https://www.ncbi.nlm.nih.gov/geo), dataset number GSE76427. This dataset contains 167 samples (cancer and paracancer), of which 115 cancer tissue expression profiles and their clinical information were used for model construction and subsequent analysis. The Cancer Genome Atlas (TCGA) liver cancer RNA-seq data were downloaded from UCSC XENA (http://xena.ucsc.edu/) to validate model genes.

**Bioinformatics analysis**

Based on the sequencing results, the differentially expressed circRNAs, lncRNAs and mRNAs were analysed. miRNAs interacting with circRNAs were predicted and screened based on the TargetScan and miRanda databases. To explore the target mRNAs of the above miRNAs, the miRecords, miRTarBase and TarBase databases were explored using the R package "multiMiR". Using the R packages "clusterProfiler", "org.Hs.eg.db", and "ggplot2", the ceRNA network was visualised using GO and KEGG. GO and KEGG functional enrichment of mRNAs in the ceRNA network. The prognostic model associated with OS was constructed based on LASSO COX regression using the R package "glmnet", and the model AUC was calculated using the R package "pROC". The risk coefficients obtained from the LASSO model were used to calculate the risk score. Patients with HCC were divided into high and low-risk groups according to the median risk score, using the R packages "survival" and "survminer-ggsurvplot " for survival analysis and visualisation, and the R package "survROC" for plotting survival ROC curves. In addition, the R package "rms" was used to construct a predictive model of the effect of genes on survival time in patients with liver cancer using Nomograms and their calibration curves. Univariate and multifactorial COX analyses were performed to explore the clinical value of risk scores as a prognostic factor for patients with liver cancer.

**Western Blot**

1、Preparation of protein samples for electrophoresis

(1) Protein collection: Discard the cell culture medium, rinse the walled cells 3 times with pre-chilled PBS at 4℃, place on ice, add 80-100ul of cell lysate, and invert on ice for 10min to cover the cell surface evenly. Use a cell spatula to collect samples from top to bottom along the bottom of the culture flask in an EP tube.

(2) Protein concentration determination: BCA protein concentration determination kit to determine protein concentration.

(3) Protein sample storage; each group of protein samples were diluted to the same concentration using the loading buffer and distilled water, boiled at 95℃ for 5min, and stored at -80℃ after the samples were cooled.

2、SDS-PAGE electrophoresis

(1) Glue filling: put the glass plate into the clip and snap it tightly and pay attention to the alignment, then snap it vertically on the shelf, use deionized water to make sure there is no leakage, absorbent paper to dry the glass plate and prepare for glue filling. According to the kit first fill the lower 10% separation gel with 5 ml gun, the height is about 2 cm from the upper edge of the glass plate (speed first fast then slow). Then the gel was slowly filled with a layer of distilled water to seal the gel. After about 20 min, it is obvious that there is a fold line between water and gum that gelatin has solidified, using filter paper to absorb the upper layer of distilled water. Then fill the upper layer of 5% concentrated gel, quickly add to the glass plate, pay attention to avoid the generation of bubbles, and then insert the comb, keep the level. 20~30min after waiting until the concentrated gel solidified, vertical upward gently pull out the comb.

(2) Configure electrophoresis buffer: weigh Tris 6.06 g, glycine 28.8 g, 10% SDS solution 20 mL, add distilled water 1000 mL to dissolve fully, and then fix the volume to 2000 mL with distilled water and store in refrigerator at 4 ℃.

(3) Assemble the electrophoresis device: select the electrophoresis device matching with the glass plate for assembly, add the appropriate amount of electrophoresis buffer to the inner tank between the glass plate, the height is level with the upper edge of the glass plate, and add the remaining electrophoresis buffer to the outer tank after confirming that there is no leakage.

(4) Sample electrophoresis: Take the previously prepared protein sample and boil it in boiling water for 5 min to denature the protein. Add protein marker and sample with micropipette and gun tip against the wall, the sample volume is 20 μL per well, and protein marker is 6ul, change the gun tip in each well during the process to avoid bubble generation and cross use of gun tip between samples. Connect the power supply and adjust the voltage to 80 V. After the blue band reaches the bottom of the glass plate, turn off the power supply and end the electrophoresis.

(5) Configure the transfer solution and PBST solution:

Membrane transfer solution: Weigh Tris 6.06 g, glycine 28.8 g, methanol 400 mL, add distilled water 1000 mL to dissolve fully, and then fix the volume to 2000 mL with distilled water.

PBST solution: Pour the PBS instant pellets into a beaker, add distilled water 1000 mL and then fully dissolve, then add 2 mL of Tween-20, and finally fix the volume to 2000 mL with distilled water and store at 4℃.

(6) Transfer membrane: first soak the required transfer clips, double-layer filter paper and liner in transfer buffer for 2~3 min, cut PVDF membrane soaked in methanol for 5 min in advance, then carefully close the clips after listing the black side of the clips, liner, filter paper, gel, PVDF membrane, filter paper and liner in order, using a test tube to drive away the air bubbles caught in them during the process, but avoid moving the position of the pads Use the test tube to remove the air bubbles during the process, but avoid moving the position of the pad. Install the clamps in the correct direction according to the instructions of the device (black side to the black side of the tank, white side to the red side of the tank), fill the tank with the transfer solution, put an ice pack into the tank, place the whole tank in crushed ice, and transfer the film overnight at 4°C in a refrigerator with 40 V. After finishing, turn off the power device to complete the transfer. The transfer clips were carefully removed and opened, and the transferred PVDF membranes were clamped out with forceps. Follow the protein Marker strip, cut the strip where the target protein of the sample is located and mark it well, remove the excess part.

(7) Immunoreactivity: After the end of membrane transfer, configure 5% skim milk powder, dip the PVDF membrane into the skim milk powder and close it on a 60r/min shaker at room temperature for 1 h. Dilute the primary antibody closure buffer with PBST, place the membrane closed by skim milk powder into an appropriate size of cling film, add the configured primary antibody closure solution, seal the membrane with a sealing machine and close it on a 60r/min shaker at room temperature for 1 h. Place the membrane flat on a 4.5 mm x 4.5 mm shaker. Closed for 1h, leveled and placed in 4℃ refrigerator overnight. Cut the cling film, carefully remove the film from the closure solution with forceps, and wash the film with PBST for 10 min on a shaker at room temperature, three times. Prepare the horseradish peroxidase-labeled secondary antibody dilution as described above, place the washed membrane into a suitable size of cling film, add the diluted secondary antibody buffer, seal the membrane with a sealing machine, and close the membrane on a shaking table at room temperature for 2 h at 60 r/min. Cut open the cling film after the secondary antibody closure, carefully remove the membrane from the closure solution with forceps, place it in a container with PBST buffer, and wash it on a shaking table at room temperature for 15 min. The membrane was washed on a bed for 15 min, 3 times.

(8) Chemiluminescence: Wear rubber gloves to configure the luminescence solution under light-proof conditions, and mix the equal volumes of liquid A and liquid B. Clamp out the membrane from the buffer with forceps, control the water and put it in the dark box, keep the marked front side up, add luminescent solution evenly to the membrane so that it is completely covered, after a few minutes visible luminescent bands, save and keep the pictures. The protein samples were added in boiling water for 5 min, denatured sufficiently, and then the target proteins were separated by SDS-PAGE electrophoresis, transferred to the membrane and sealed. Add diluted primary antibody, incubate overnight at 4℃, wash the membrane, add horseradish peroxidase-labeled secondary antibody dilution, incubate for 2 h at room temperature and wash the membrane. Add chemiluminescent solution, and the luminescent bands will be visible after a few minutes, save and keep the pictures.

**Real-time PCR**

1. PCR primer design

Firstly, we logged into the NCBI homepage (http://www.ncbi.njm.nih.gov/) and searched for Gene IDs. We logged into the primer design page PrimerBank (https://pga.mgh.harvard.edu/primerbank/) and entered the Gene IDs obtained from NCBI to obtain the primer sequences.

2. RNA extraction and quantification

(1) After aspirating the cell culture solution, wash it twice repeatedly with PBS, add 1ml Trizol (2*10^6^ cells/200mg tissue) to lyse for 2min, pipette and blow down the cells evenly and transfer into EP tube and leave it on ice for 5min.

(2)Add 0.2ml of chloroform, mix upside down 10 times, and centrifuge at 12000r/min, 4℃ for 20min after standing on ice for 5min.

(3) Pipette the supernatant into another EP tube, add the same volume of isopropanol, mix upside down 10 times, let stand on ice for 10 min, then centrifuge at 12000r/min, 4℃ for 15 min.

(4) Pipette the supernatant carefully and discard, then add pre-cooled 75% ethanol to the EP tube and centrifuge at 12000r/min, 4℃ for 10min.

(5) Discard the supernatant by pipetting, dry the EP tube upside down at room temperature for 5 min, and add 20 μL of DEPC water to the EP tube (place it at -80℃ for backup);

(6) Quantification by UV spectrophotometer with micro quartz cup, first use 100ul of deionized water as blank control, blot dry on blotting paper and then put on sample for detection, select samples with OD260/280 between 1.8~2.0.

3、Reverse transcription

(1)1ul Oligo (DT)18 plus 5 μg RNA, make up to 12 μL with DEPC treated water, place at 70℃, 5min, take out and place on ice quickly;

(2) Add 4 μL of 5* buffer, 1 μL of RNase inhibitor and 2 μL of 10mmol/L NTP, mix well and place on ice at 37°C for 5min;

(3) Add 1 μL M-MulV reverse transcriptase, reverse transcribe for 60min at 42°C and terminate the enzyme activity for 10min at 70°C;

(4) 20 μL of reverse transcription product was stored at -20℃.

4、PCR

(1)Configure PCR system in PCR tube and mix well: 25*SYBR Green I Master Mix 12.5 μL, primers 100~1000 nmol/l, cDNA 10~50 ng, make up deionized water to 25 μL.

(2) Centrifuge at 1000rpm, room temperature, for 5min;

(3) After setting the PCR instrument, place the PCR tube, the first step 95℃, 2min; the second step 95℃, 10s; the third step 60℃, 30s, complete 40 cycles, at 60℃, set the fluorescence detection point for amplification.

The reaction sequence was as follows:

Primer sequences

| Target genes | Category | Primer sequences(5’- 3’) |
| --- | --- | --- |
| CD133 | Forward | GCAGCAGTCTGACCAGCGTGAA  ACGGGTGGAAGCTGCCTCAGTT |
|  | Reverse |  |
| CD90 | Forward | ATCGCTCTCCTGCTAACAGTC |
|  | Reverse | CTCGTACTGGATGGGTGAACT |
| Circ-TAX1BP1 | Forward | TTGCAAAAATGGAGCTGAAA  GATGGGCTTTGGATGTAGCA |
|  | Reverse |  |
| Circ-ZEB1 | Forward | CACCTGAAGAGGACCAGAGG  CCACAATATGCAGTTTGTCTTCA |
|  | Reverse |  |
| Circ-PHC3 | Forward | TGTCACCCGGACATCAAGTA  GGGTAATACTGCCGCTGGTA |
|  | Reverse |  |
| Circ-AFAP1 | Forward | GAAGGACCATGCTCAGAAGC  CTGCCTTTTTCTCCCTGACA |
|  | Reverse |  |
| Circ-CCNB1 | Forward | TGGTGCACTTTCCTCCTTCT  AACATGGCAGTGACACCAAC |
|  | Reverse |  |

**Immunohistochemistry**

(1) Prepare experimental reagent items and instruments such as tissue microarrays, antibodies, antigen repair solution, IHC kits, immunohistochemistry pens, coverslips, vertical pressure steam sterilizers, and electric thermostatic blast drying ovens required for the experiments.

(2) Set the electric thermostatic blast drying oven to 65℃, and bake the wax-sealed tissue samples in the drying oven for 3h.

(3) Dewaxing and hydration: The baked tissue samples were dewaxed and rehydrated in the following order in the following order over the cylinder, each time the cylinder was placed: 3 xylene for 15 min each, 100% ethanol for 10 min each, 90% ethanol, 80% ethanol, 70% ethanol for 5 min each. after completing the above steps the slices were rinsed under running water for 3 times, each time for 5 min.

(4) Antigen repair: Remove the tissue chip after water, put it into the box with antigen repair solution, put the box into the autoclave i.e. vertical pressure steam sterilizer, cover the lid, set the temperature to 120℃ and start working. When the pressure of the autoclave reaches 120℃, time 2 min, turn off the switch when time is up, carefully lift the autoclave gas valve to deflate, cool at room temperature, and open the autoclave to remove the slice box after the pressure gauge pointer drops to 0.

(5) After the antigen repair solution in the cassette cools to room temperature, wash the sections with PBS solution 3 times for 5 min each time, then put the sections into 3% hydrogen peroxide solution and leave them at room temperature for 10 min.

(6) Closure: Wash the sections 3 times with PBS solution for 5 min each time. then shake off the water stains on the slices, draw water-blocking circles with an immunohistochemical pen, add 5% BSA solution dropwise to the surface of the sections inside the water-blocking circles, and close them in the wet box for 1 h.

(7) Incubate the primary antibody: remove the slice, shake off the liquid on the slice, and dry the drop plus residual water with laboratory paper, taking care not to touch the tissue. Prepare the primary antibody liquid with 5% BSA solution at a dilution ratio of 1:100, about 300 μL of liquid is required for each tissue microarray. add the primary antibody dropwise to the tissue surface and incubate the sections in the wet box at 4°C overnight.

(8) Incubate the secondary antibody: remove the wet box and sections on the next day, let them recover to room temperature naturally, shake off the antibody on the sections, wash them with PBS solution three times for 5 min each time, shake off the water on the sections, dab the excess liquid with paper, add the secondary antibody working solution in the water blocking circle, put them in the wet box, and incubate the secondary antibody for 20 min at room temperature and avoid light.

(9) Incubate the triple antibody: remove the slice, wash with PBS solution 3 times for 5 min each time. add the triple antibody and incubate for 20 min, rinse with PBS 4 times for 5 min each time.

(10) Color development: Dilute DAB staining solution with 1:20 ratio of double-distilled water, add DAB staining solution dropwise to the slices, observe the degree of color development under the microscope during the period, and adjust the color development time according to the color development effect.

(11) Re-staining: Immediately after successful color development, the sections were immersed in water to terminate the color development reaction, washed once with water and rinsed once with distilled water. Remove and shake the slices dry, immerse the slices into filtered hematoxylin staining solution and time for 5 min. rinse with running water, the staining time can be adjusted according to the staining results. Place the stained slices into 1% hydrochloric acid alcohol for 3 times and rinse under running water.

(12) Dehydration: Place the sections in the following solutions in turn: 85% ethanol, 95% ethanol and anhydrous ethanol for 3 min each and 2 xylenes for 5 min each.

(13) Remove the sections and lay them flat, and seal them with neutral resin when they are half dry. After the sections were dried, they could be observed and photographed under the light microscope.

(14) Analysis of results: IHC staining score = staining depth × percentage of positive cells. Staining intensity score: staining intensity score: colorless (0 points), light yellow (1 point), brownish yellow (2 points), yellowish brown (3 points). Percentage of positive cells points: 0-5% (0 points), 6%-25% (1 point), 26%-50% (2 points), 51%-75% (3 points), 76%-100% (4 points). Finally, the IHC staining score was based on the product of the two. Immunohistochemical staining results were evaluated and examined independently by two senior pathologists.

**Overexpression lentivirus**

The overexpression lentiviruses LVKL84495-1 (OE-circ-ZEB1) and LVKL84496-1 (OE-circ-AFAP1), and the negative control virus LVCON254 (NC), were purchased from Gikai Genetics, China.

(1) The virus plasmids were removed from the -80℃ refrigerator, slowly melted on ice, and the viruses were appropriately diluted. According to the results of the pre-experiment, the cells were best when MOI=20, the initial number of cells per dish was 1.25*10^6^, the number of infected dishes was 4 dishes, and a total of 1*10^8^ TU virus was used, TU=total virus dosage, MOI=(virus titer×virus volume)/number of cells;

(2) Carefully remove the 6-well plate from the incubator, and after microscopic observation of the cell status, disinfect with 75% alcohol spray and place in a biosafety cabinet, discard the supernatant of each well, wash twice with PBS, replace with new complete medium without antibiotics, add diluted lentiviral plasmids (OE-circ-ZEB1, OE-circ-AFAP1, NC) and infection enhancing solution, and continue to culture;

(3) After 12h of incubation in CO_2_ incubator at 37℃, the culture was continued with new complete medium. If, during this period, cell morphology changes occur, the change of medium can be advanced to 8h;

(4) 72h after infection, observe the cell infection efficiency under fluorescence microscope.

**Stable transfected cell lines**

(1) After transfected cells were passaged, they were inoculated into 6-well plates, and the cell density was adjusted according to the cell status to 24h The cell growth density was adjusted to approximately 70%, and the cell line was screened by adding puromycin (2 μg/mL). The cell lines were screened by real-time PCR and Western blot to detect the expression of circ-ZEB1 and circ-AFAP1 to judge the transfection effect;

(2) After transfected cells were passaged, they were inoculated into 6-well plates, and the cell density was adjusted according to the cell status to approximately 70% of the cell growth density at 24 h. The cell lines were screened by adding puromycin (2 μg/mL). The expression of circ-ZEB1 and circ-AFAP1 was detected by real-time PCR and Western blot to determine the transfection effect;

(3) The culture was continued and the culture medium contained puromycin was changed every 3-4 days until cells in the blank control wells that were not transfected with plasmids were completely dead;

(4) The culture medium was reduced to a maintenance concentration of puromycin (0.5-1 μg/mL), and the transfected cells were continued to be screened and amplified. The cells were also collected for real-time PCR and Western blot assay to identify the expression of circ-ZEB1 and circ-AFAP1, and the cells with normal results were frozen and stored.

**Molecular Docking**

Download SDF format files of Cabozantinib, Donafenib, Lenvatinib, Regorafenib and Sorafenib 2D structures from PubChem database, convert them into 3D structures by ChemBio3D Ultra 14.0 software and save them as mol2 format files.CYP1B1, PIK3R1, POFUT2, PRKAG1, SETDB1, SKAP2, TRAF2, ZDHHC17 protein crystal structures (BTBD9, CEP89, GATAD1, RHOBTB3 RPRD1A, SPI1, TRIM35, YIPF4 were not found), and then imported into Pymol software for de-watering and de-hybridization, and saved as pdb format files. Use AutoDockTools-1.5.6 software to obtain pdbqt format files of small molecule ligands and protein receptors, and determine the structure center of protein receptors and set docking parameters. Molecular docking was verified using vina software, and docking results with large absolute values of binding energy (affinity) were visualized using PyMOL software.

***Separation of CSV-positive and CD90-positive cells***

*Collection of T25 cell culture flasks (70-80% confluency, cell count 5×105), wash once with 1×PBS, incubate with 1mM EDTA for 10 minutes (CD90 group digests cells with trypsin). Gently tap the cells down into a centrifuge tube using 10% DMEM medium, centrifuge at 1000 rpm for 5 minutes, discard the supernatant. Add 100ul of Easy Buffer to resuspend the cells, to reduce non-specific binding, add 20μl of FcR blocker to the cell suspension, gently flick for 60 times to thoroughly mix the reagents and cells. Perform separate screening for CSV positive cells and CD90 positive cells. Label the cells with CSV and CD90 antibodies respectively, incubate on ice for 20 minutes. Add 5ml of MACS Buffer for washing, centrifuge at 700g for 4 minutes, discard the supernatant. Resuspend the cells with 100ul of MACS Buffer, add Anti-Mouse Beads to the cell suspension, incubate on ice for 20 minutes, add 5ml of MACS Buffer for washing, centrifuge at 700g for 4 minutes, discard the supernatant. Resuspend the cells with MACS Buffer, perform separation using LS columns, obtain separate populations of CSV positive and CD90 positive tumor cells, as well as CSV negative and CD90 negative tumor cells.*

***Cellular immunofluorescence***

*Count both CSV positive and CSV negative cells. Add 100μl of MACS Buffer and incubate with CSV antibody (incubation at 37℃ in a CO2 incubator for 1 hour). Open the cytospin machine and place a filter paper and a glass slide. Centrifuge at 700g for 4 minutes. After centrifugation, open the cytospin chamber. Draw a black circle on the back of the glass slide. Use an immunohistochemistry pen to draw a circle along the black line on the front of the glass slide. Fix with 4% PFA for 10 minutes and wash with PBS three times. Incubate with 2.5% DAPI for 5 minutes, followed by three washes with PBS. Finally, observe and capture images under a fluorescence microscope.*

**Statistical analysis**

All computational analyses and graphical visualisations were carried out using Prism (Graph Pad Prism 8) and R statistical software (Cran R Project R program v3.6.0). Two or more groups of continuous variables were compared using parametric tests (Student's t-test or ANOVA) or non-parametric tests (Wilcoxon rank sum test or Kruskal-Wallis test). Correlation between two continuous variables is measured by Pearson's r correlation or Spearman's order correlation. Survival analysis was performed using the Kaplan-Meier method, and the log-rank test was used to determine the statistical significance of differences. P-values less than 0.05 were considered statistically significant.
